# Fulminant amoebic colitis in the era of computed tomography scan: A case report and review of the literature

**DOI:** 10.4102/sajr.v22i1.1354

**Published:** 2018-08-15

**Authors:** Suman Mewa Kinoo, Vikesh V. Ramkelawon, Jaynund Maharajh, Bugwan Singh

**Affiliations:** 1 Department of Surgery, University of KwaZulu-Natal, King Edward VIII Hospital, Durban, South Africa; 2 Ethekwini Hospital and Heart Centre, Durban, South Africa; 3 Department of Radiology, University of KwaZulu-Natal, King Edward VIII Hospital, Durban, South Africa

## Abstract

Amoebic colitis, caused by ingestion of water or food contaminated with the protozoan Entamoeba histolytica, can progress to a fulminant colitis. Computed tomography (CT) findings reported in the literature on this type of colitis are sparse. We present a 59-year-old male patient with a one-week history of progressive abdominal pain, abdominal distension and associated watery and bloody diarrhoea. A CT scan revealed deep ulcerations with submucosal and intramural tracking of contrast. Colonoscopy and biopsy confirmed a diagnosis of Amoebic colitis. The patient required a laparotomy and demised. Deep ulcerations with submucosal and intramural tracking of contrast on CT are diagnostic of fulminant amoebic colitis. Although not demonstrated at CT in this case, discontinuous bowel necrosis, omental wrapping (seen at laparotomy in our case) and neovascularisation of the bowel wall may be other features to look out for.

## Introduction

Fulminant amoebic colitis (FAC) is the transmural extension of amoebic colitis, associated with a devastating outcome. Until a few decades ago, the diagnosis of FAC was made without the recourse to computed tomography (CT) scan; diagnosis was largely clinically supported by stool examination and sigmoidoscopic examination, with plain radiology and sonography having a subsidiary diagnostic role.

In current practice, largely because of improvements in sanitation and primary health care access, FAC is uncommon; thus, the recognition of this entity may be found to be lacking, but should be suspected in those vulnerable to sexually transmitted disease, human immunodeficiency virus infection and those travelling from endemic areas. The difficulty in diagnosis is offset by improvements in diagnostic modalities and their availability, as prompted by our recent experience in managing a patient with FAC. To this end, the role of CT scan in the diagnosis of FAC merits review.

## Case history

**FIGURE 1:**
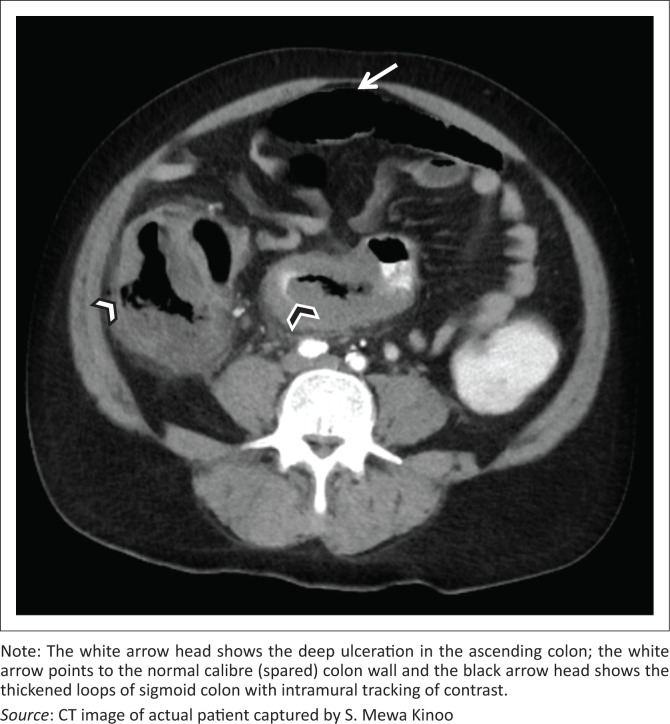
Computed tomography scan with triple contrast.

**FIGURE 2:**
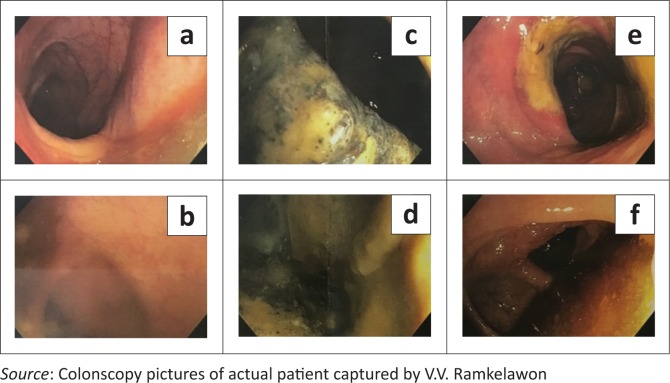
Colonoscopy findings: Areas of normal mucosa (a and b), necrotic mucosa (c and d) and inflamed mucosa (e and f).

**FIGURE 3:**
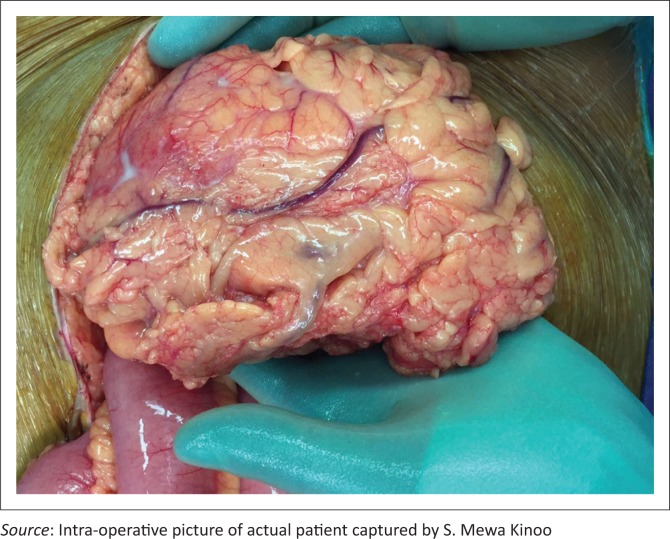
Intra-operative picture of the omental ‘wrap’ involving the caecum and ascending colon.

**FIGURE 4:**
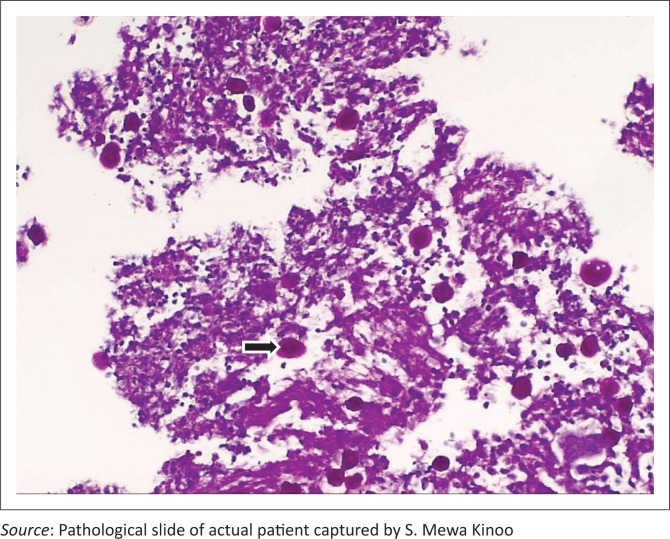
Multiple round to oval amoebic trophozoites (black arrow) measuring 6 nm – 40 nm, resembling macrophages, are seen lying freely within debris.

A 59-year-old male patient presented with a one-week history of progressive abdominal pain, abdominal distension and associated watery and bloody diarrhoea. There was no history of inflammatory bowel disease, nor antibiotic or herbal enema usage; the patient reported drinking only tap water. On examination, the patient, who was HIV-negative, was dehydrated, pale, pyrexial and had a tachycardia. The blood pressure was normal. The abdomen was distended with generalised tenderness but no peritonism. Rectal examination revealed blood on the glove. The patient was resuscitated and commenced on intravenous antibiotics as an infective cause for his acute abdomen was considered most likely. Plain chest radiography revealed no pneumoperitoneum, and abdominal radiography revealed a transverse colon diameter of 7 cm with neither thumb printing nor pneumatosis intestinalis. The white cell count was 34 × 10^9^/L, haemoglobin 9 g/dL, urea 9 mmol/L and creatinine 100 µmol/L. An abdominal triple contrast-enhanced (oral, rectal, intravenous) CT scan revealed segments of grossly dilated large bowel with areas of thickening, deep ulceration, contrast tracking deep within the bowel wall and skip areas with normal bowel wall calibre (Figure 1), suggesting colitis. Colonoscopy revealed areas of inflamed and necrotic mucosa with intervening areas of normal mucosa (Figure 2). Following colonoscopy and biopsy, the patient’s condition deteriorated and a post-colonoscopy perforation was suspected. At laparotomy, the entire colon was noted to be wrapped with omentum, with no signs of perforation or peritonitis (Figure 3). A diagnosis of amoebic colitis was entertained because of the omental wrapping. A diverting loop ileostomy was fashioned as the patient was haemodynamically unstable. Anti-parasitic treatment was commenced immediately post-operatively; however, despite this, the patient deteriorated and demised. Histopathological evaluation of the colonoscopically guided biopsy confirmed the diagnosis of amoebic colitis (Figure 4).

## Discussion

Amoebic colitis is caused by ingestion of water or food contaminated with the protozoan *Entamoeba histolytica (E. histolytica*). Once the ingested cysts reach the terminal ileum, they undergo excystation and release the trophozoite.^1^ The trophozoites may or may not penetrate the colonic mucosal barrier. If no penetration occurs, this is regarded as a non-invasive form and the patient has no luminal pathology and no symptoms; if mucosal penetration occurs, the patient will develop symptoms ranging from mild colitis (superficial ulcers, abdominal pain, secretory diarrhoea) to the devastating FAC (associated with deep ulcers, perforation and faeculant peritonitis) which carries a high morbidity and mortality in the range of 55% – 100%.^2,3^

Whereas mild amoebic colitis may be diagnosed and treated effectively utilising conservative measures, the confirmation of FAC on the basis of diagnostic modalities may be challenging. Historically, amoebiasis was confirmed on stool microscopy identifying trophozoites and cysts or stool antigens and the detection of antibodies specific for *E. hystolytica*.^4^ Because of the low sensitivity of these non-invasive tests, they have been replaced by molecular detection using polymerase chain reaction that demonstrates excellent sensitivity in identification of symptomatic and asymptomatic stool carriers.

In addition to the demonstration of free intraperitoneal air or retroperitoneal mottling in patients with FAC, the plain abdominal radiograph may reveal features distinctive to amoebic colitis. Classically, the involved segments of the colon are not visualised (similar to toxic megacolon of ulcerative colitis) and gaseous distention occurs in the non-diseased colon. In addition, Luvuno described ‘fixed displacement’ of the colon as an indicator of the site of perforation, as the uninvolved colon acts as a patch over the perforated areas, to which it becomes adherent.^5^

Although generally not impacting considerably on the overall diagnosis of amoebic colitis, ultrasound examination of the liver is important as between 20% and 30% of patients with amoebic colitis have an associated amoebic liver abscess; recognition and treatment of the latter enhance the overall clinical outcome.

Invariably, the diagnosis of amoebic colitis may have to be confirmed by a procto-colonoscopy with biopsy demonstrating *E. histolytica* and the pathognomonic flask-shaped ulcers. However, this carries the potential risk of perforation, especially in an acutely inflamed and possibly ischaemic colon. Furthermore, as in this case report, results of the various diagnostic tests may not be timeously forthcoming for a definitive diagnosis and a recourse to laparotomy may be required.

Although colitis caused by cytomegalovirus, salmonellosis, ulcerative colitis, pseudomembranous colitis and ischaemic colitis may present with pancolitis, discontinuous bowel necrosis is extremely rare in these types of colitis. Furthermore, as a result of the ischaemic phenomenon in amoebic colitis, the omentum forms adhesive ‘wraps’ that afford both mechanical protection from microperforation and neovascularisation of the diseased colon, a phenomenon described by Luvuno.^6^ This phenomenon may be apparent on CT scan imaging.

Even if a diagnosis of FAC is made, the clinical challenge is whether to perform surgery on a severely ill patient, because there may be a role for effective conservative treatment when colonic perforations are contained by the omental ‘wraps’ and, less so, by the unaffected small bowel. The role of CT scan in identifying the latter category of patient would be appropriate.

The decline in the incidence of FAC affords little opportunity to correlate this diagnostic modality with the features of FAC, although there are reports of non-specific CT scan features for FAC.^7^ Thus, the literature describing the CT scan features of FAC is sparse.

In a case report, Ying et al. report extended submucosal ulcers with intramural dissection of contrast as the characteristic CT scan finding of FAC.^8^ This feature (noted in our patient) represents the underlying flask-shaped ulcer pathology typical of amoebiasis and excludes other infective, inflammatory or ischaemic bowel diseases (Figure 1). Other non-specific findings (not noted in our patient) include the target sign indicating pancolitis and discontinuous bowel necrosis depicted by alternating enhancing and non-enhancing bowel wall. The latter confirms the vascular occlusion associated with FAC.^9^

Contrary to the role of CT scan in the diagnosis of FAC, this modality is increasingly used as the primary imaging modality for ulcerative colitis and Crohn’s disease. Although there is a considerable overlap between the CT scan findings of ulcerative colitis and Crohn’s disease, certain defining features of each disease have characteristics that afford distinction from FAC.

Ulcerative colitis is predominantly a mucosal disease, but with disease progression, there is bowel wall thickening that is less than that seen in Crohn’s disease. The CT scan features of ulcerative colitis include circumferential, symmetrical thickening of the bowel wall up to 8 mm with fold enlargement (maximal thickness of normal colonic wall is 3 mm when distended and 5 mm when collapsed). Furthermore, there is a target appearance of the rectum with proliferation of the perirectal fat.

Crohn’s disease has transmural involvement that progresses to replacement of submucosal fat with fibrosis and loss of mural stratification.^10^ Crohn’s colitis is also associated with a greater colon wall thickness (> 10 mm) that is eccentric, preferentially mesenteric, and segmental with skip lesions. In addition, there is an association with several extraluminal features such as fistula and abscess formation and mesenteric abnormalities.^10^

The role of CT in the evaluation of Crohn’s disease is well accepted. The ability of CT to depict bowel involvement and extraluminal pathology (e.g. abscess, obstruction and fistula) makes it an essential imaging tool for patient care. The earliest CT finding of Crohn’s disease is bowel wall thickening, which usually involves the distal small bowel and colon, although any segment of the gastrointestinal tract can be affected.^10^ Typically, the mural thickening is 5 mm–15 mm.^11^ A misdiagnosis of FAC as Crohn’s disease can be deleterious for the patient if steroid treatment is implemented.^3^

## Conclusion

The challenges presented by FAC are confirming the diagnosis (which may require a colonoscopy and biopsy, with its inherent risks of perforation) and deciding on the appropriate management. Management depends on the severity of the colitis; both mild and severe colitis can be successfully treated conservatively; however, operative intervention is required when there is a free or a sealed perforation, contained by an omental wrap, which both progress to transmural disease, manifesting as FAC. Correlating the CT scan findings with the pathological manifestations of the FAC will prompt an appropriate management.

Although a vast differential diagnosis of colitis should always be kept in mind, the majority of other colitis conditions such as ulcerative colitis, inflammatory colitis and ischaemic colitis can be excluded. Crohn’s colitis and cytomegalovirus (CMV) colitis may mimic amoebic colitis because of the skip lesions, but these conditions do not present with deep ulcerations, submucosal and intramural tracking of contrast, discontinuous bowel necrosis or omental wrapping and neovascularisation of the bowel wall.
